# The burden of depressive disorders in musculoskeletal diseases: is there an association between mood and inflammation?

**DOI:** 10.1186/s12991-020-00322-2

**Published:** 2021-01-04

**Authors:** Maria Sole Chimenti, Giulia Lavinia Fonti, Paola Conigliaro, Paola Triggianese, Emanuela Bianciardi, Marialuce Coviello, Ginevra Lombardozzi, Giulia Tarantino, Cinzia Niolu, Alberto Siracusano, Roberto Perricone

**Affiliations:** 1 Rheumatology, Allergology and Clinical Immunology, Department of Systems Medicine, University of Rome Tor Vergata, Rome, Italy; 2 Psychiatric Chair, Department of Systems Medicine, University of Rome Tor Vergata, Via Montpellier 1, Rome, Italy

**Keywords:** Major depression, Mood disorders, Rheumatoid arthritis, Psoriatic arthritis, Spondyloarthritis, Biological therapies, Obesity

## Abstract

**Importance:**

Evidence emerged concerning how inflammatory arthritis and mood disorders can often occur in the same patient and show a similar clinical pattern. An overview of the rheumatological and psychiatric aspects of these diseases can certainly be useful for the improvement of patients' clinical and therapeutic management.

**Objective:**

The aim of this narrative review was to summarize existing literature about common pathogenetic and clinical aspects as a means of improving management and therapeutic approach in patients affected by rheumatoid arthritis, psoriatic arthritis and spondyloarthritis. Outcomes such as disease activity indexes and patient reported outcomes (PROs) were considered.

**Findings:**

Common pathogenetic pathways emerged between inflammatory arthritis and mood disorders. Pro-inflammatory mechanisms, such as TNFα, IL-6, IL-17 and oxidative stress factors as well as neurotransmitter alterations at the level of CNS and blood–brain barrier (BBB) cells are involved. The activation of these common pathogenetic pathways is, also, affected by the same triggers, such as smoking, stress, lifestyle, and evidence has emerged concerning the possibility of the clinical efficacy of using the same therapeutic approaches.

**Conclusions:**

The main causes of the variability in clinical studies outcomes are the rheumatological diseases considered, the prevalence of depression in the general population and in patients with rheumatological diseases and the type of depressive symptom examined. Patients affected by inflammatory arthritis can present symptoms and signs in common with mood disorders, leading to possible clinical overlap. There are still few studies analyzing this concept: they are extremely heterogeneous, both in the characteristics of the population taken into consideration and in the methods used for the definition of depressive disorder, but the suggestions of the data obtained so far are promising and deserve to be pursued.

## Introduction

Rheumatic diseases are a group of disorders mainly characterized by joint pain. Among them, rheumatoid arthritis (RA), psoriatic arthritis (PsA) or spondyloarthritis (SpA) are considered systemic diseases because of the presence of both articular involvement and systemic features with related comorbidities [1, 2]. RA is a chronic autoimmune systemic inflammatory disease that may involve many organs and tissues, but, principally, affects the peripheral joints in a symmetrical pattern. Worldwide, it represents the most common inflammatory arthropathy [2]. RA is characterized by the presence of autoantibodies such as rheumatoid factor (RF) and anti-citrullinated protein antibodies (ACPA) [3]. On the other hand, SpA are a heterogeneous group of inflammatory chronic diseases characterized by sharing common pathogenic, clinical and radiologic features, typically defined as “seronegative” due to the absence of RF. However, recent evidence supported the presence of autoimmunity in SpA. Evidence demonstrated the presence of autoantibodies in serum of SpA patients, revealing and supporting the autoimmune features of the disease [4]. Both RA and SpA patients often present comorbidities related to mood disorders, but the evaluation of their prevalence, as yet remains a matter for discussion. As demonstrated by Matcham et al. [5] in their meta-analysis conducted on a total of 72 studies, made up of 13,189 patients, prevalence estimates for depression in RA patients range between 9.5% [6] and 41.5% [7]. This high variability may be related, first of all, to the criteria of depression diagnosis used in the individual studies and secondly to the multitude of psychometric tools available to detect depression: so far, depression is defined in 40 different ways [8]. Methods for depression evaluation take into consideration some signs and symptoms, such as insomnia and asthenia, which are also part of the spectrum of symptoms and signs presented by patients affected by RA. Matcham et al*. *estimate, from the small number of studies using gold standard clinical interviews, that major depression is present in 16.8% of RA patients, principally, female and older adult patients. The data shown by Matcham are, largely, applicable to all other autoimmune diseases, such as systemic lupus erythematosus (SLE) [9], both in terms of frequency range and in terms of confinement. For example, patients affected by PsA are supposed to be affected by depression with a prevalence rate of between 19 and 62% [10], whereas in the larger group of SpA it ranged from 11 to 64% [10] owing to the same reasons listed for RA.

Furthermore, the assessment of the depressive symptoms prevalence in patients with SpA is complicated by the not so easy task of distinguishing ankylosing spondylitis (AS) and non-radiographic-axial SpA (nr-axSpA) [11].

A close pathogenetic relationship has been hypothesized between chronic inflammatory diseases and depression [12]. Depressive symptoms, such as depressed mood and asthenia, are responsible for a high impact in societal burden, especially in patients already suffering from chronic diseases, like RA and SpA [13]. These symptoms are frequently identified in patients affected by joint diseases presenting disabling symptoms, which affect their quality of life and productivity. Signs and symptoms are summarized in Table 1. It is intriguing as both autoimmune diseases, like RA and SpA, and mood disorders, like major depression, may have similar pathogenetic pathways and immunological activations. Moreover, it has been previously demonstrated that treatments for depression supported this immuno-psychiatric link: antidepressants have been shown to decrease inflammation, whereas, for most treatments, higher levels of baseline inflammation predicted lower treatment efficacy [14]. Patients with autoimmune diseases like RA may, on the other hand, have benefits from immunomodulatory treatments, not only for the joint disease but also for depressive symptoms, supporting the hypothesis that depression and asthenia are associated with an increased activation of the innate and acquired immune system, which may serve as a valid target for treatment [15].

**Table 1:** Characteristic symptoms and signs of inflammatory arthritis and depressive syndromes

Symptoms	Depressive disorders	Rheumatoid arthritis	Psoriatic arthritis	Spondyloarthritis	Fibromyalgia
Fatigue	√^13^	√^78^	√	√	√^87^
Asthenia	√^13^	√^78^	√	√	√^87^
Stiffness	–	√^78^	√	√	
Poor sleep	√^13^	√^78^	√	√	√^87^
Chronic pain	√^13^	√^2^	√^82^	√	√^87^
Malformations	–	√^2^	–	√^80^	
Daily disability	√^13^	√ ^2^	–	√^80^	√
Work inability	√^13^	√^2^	–	√^80^	√
Impaired relationships	√^13^	√^2^	√^82^	√^80^	√^87^
Sexual dysfunction	^13^	–	√^82^	√^80^	√^87^
Shame	–	–	√^82^	–	–
Guilt	–	–	√^82^	–	–
Public Embarrassment	–	–	√^82^	√^74^	–
Suicidal ideation	√^13^	–	√^83^	–	–

√, present and described

The purposes of this narrative review are: (i) to highlight the role of inflammation as a link between inflammatory joint diseases and depression; (ii) to discuss risk factors for depression in patients affected by rheumatological diseases; (iii) and to assess the effects of biological DMARDs (bDMARDs) on mood disorders in patients with rheumatological diseases.

The overarching aim of this review is to summarize common pathogenetic and clinical aspects of inflammatory joint diseases, as RA, PsA and SpA, and mood disorders, as major depression, considering outcomes as disease activity indexes and patient reported outcomes (PROs). There is the need to deepen the comprehension on rheumatological and psychiatric aspects of inflammatory joint diseases for the improvement of patients' clinical and therapeutic management. The close relationship between joint diseases, psychiatric disorders and lifestyles were also explored.

### Pathogenesis

Rheumatological diseases, such as Ra and SpA, may be strongly associated with the development of alterations in the patient cognitive–behavioral sphere, in particular with the development of depression [14, 15]. In this context, RA is one of the most widely studied diseases [16]. Depression is the mental health disorder most associated with RA, probably due to increased pain, fatigue, reduced health-related quality of life, increased levels of physical disability and increased health care costs. However, the causes that may influence the development of depression in patients with chronic rheumatological diseases are still under investigation**.** Hypotheses are suggested in the next paragraph.

### Inflammation, disease activity and lifestyle

The association between autoimmune diseases and depressive symptoms has been known for years [17] and numerous studies have been carried out to better understand this link. First of all, a possible role of pro-inflammatory cytokines, the main actors in autoimmune processes has been suggested in the development of CNS manifestations. Pro-inflammatory cytokines could represent the key element in combining the worlds of neuropsychiatry and rheumatology. A decisive role, in this area, is played by Th17 cells, IL-6 and TNF [18]. Th17 cells are a sub-population of CD4^+^ T cells and part of the adaptive immune system producing the inflammatory cytokine, as interleukin (IL)-17 (both IL-17A and IL-17F) [19]. Among them, IL-17A is rigorously related to the pathogenesis of rheumatological diseases, such as SpA, and, at the same time, has been shown to be capable of reducing the expression of tight junction (TJ)-associated genes and disrupted monolayer integrity in the BBB cell, promoting the passage of pro-inflammatory cytokines [20]. This process may result in an exacerbation of neuroinflammation and synaptic dysfunction. The overall effects of IL-17A produced by CNS resident cells seem to be localized [21] and not sufficient to induce depression, however it has been hypothesized that in some diseases, such as SpA, where the production of IL-17A is increased by a pathogenic sub-population of Th17 at tissue and enthesis level, making the *stimulus* working in promoting the onset of depressive symptoms [22].

Furthermore, Th17 cells differentiation is promoted by a variety of other cytokines, such as TNFα and IL-6, whose production is physiologically increased as a result of psychosocial stressors, such as negative life events and chronic psychosocial stress, which often precede the onset of clinical depression [23]. Moreover, as explained by Dantzer et al. [24], the pro-inflammatory cytokines produced by innate immune cells during influenza and during autoimmune diseases are the same. The “sickness behavior”, as it is called, understood as irritability, loss of interest in physical and social environments and fragmented sleep, connects both groups of diseases since they are caused by the same pro-inflammatory molecules. As example, in the case of flu, these symptoms tend to be underestimated because they are considered as a self-limiting situation, while in rheumatological diseases, especially in the active phase, it tends to become chronic with a high impact on patients’ quality of life and, therefore, this behavior is often linked to the sphere of depressive disorders [25–27]. Moreover, some of these symptoms, such as sleep deprivation, result in impairments in immune function, characterized by increased levels of c-reactive protein (CRP), TNF and IL-6 [28], creating an endless vicious cycle.

The effect of TNF and IL-6, in addition to increasing Th17 cell production, would seem to act through two other mechanisms:

- Negative modulation of serotonergic neurotransmission due to degradation of tryptophan [29]. This is an essential amino acid that is required as a precursor for serotonin synthesis. During inflammation, TNF is able to induce an increase in serum enzymes responsible for the degradation of tryptophan, with a negative effect on the production of serotonin and the serotonergic neurotransmission.
- Hyperactivation of hypothalamus–pituitary–adrenal axis attributes to hyperactive corticotropin-releasing factor (CRH) [30]. Pro-inflammatory cytokines promote expression of the β isoform of the glucocorticoid receptor, which results in a decreased inhibitory feedback on CRH by glucocorticoids.

It, therefore, emerged that the inflammatory process in autoimmune rheumatological diseases, as well as the periods of re-exacerbation of the disease itself, can be the basis for the development of mood changes due to neurotransmission alterations. At the same time, acute phases and relapses of disease and the chronic structural damage that consequently can develop, are responsible for negative changes in a patient's lifestyle. Moreover, obesity, smoking and eating habits, vitamin D deficiency and reduced physical activity are all elements that can accentuate the inflammatory processes and facilitate the development of depression [31, 32].

Many studies, currently, correlate diet to inflammatory status. One of them, performed by Fung et al. [33], underlined how a Western dietary pattern is associated with higher levels of CRP, while the Mediterranean diet pattern is characterized by a lower inflammatory status [34, 35]. The reason is that the fiber content and the type of fatty acids that characterize the two different diets may support a different inflammatory status. The high content of fibers present in the Mediterranean diet positively influences the intestinal microbiota, which is known to be the basis for immune dysregulation and provides a high amount of beta-glucans that promote the immune system functionality [36, 37]. As for fatty acids, a diet rich in omega-3 fatty acids, contained in seafood, nuts, legumes and leafy green vegetables, appear to have a positive impact on immune functioning [38], while omega-6 fatty acids used in processed foods, increases the production of pro-inflammatory cytokines [39]. Finally, an incorrect diet regime, can lead to a deficiency of oligo-elements and essential vitamins, such as vitamin D. Vitamin D has a well-documented modulatory effect on immune responses and in its form of 25-dihydroxyvitamin D3 (calcitriol) reduces concentrations of inflammatory markers, including TNF, CRP, IL-6 and oxidative stress markers [40]. Furthermore, reduced physical activity and obesity are additional risk factors in the development of depression and inflammation flares [41, 42].

Obesity, itself, is considered as an inflammatory state: the abdominal adipose tissue acts as a cytokines pool *reservoir*, able to produce TNF, IL-6, IL-8 and leptin [43, 44]

At the same time, sarcopenia linked to the abundance of fatty cells, leads to a high level of CRP [45]. On the other hand, regular exercise down-regulates systemic inflammation and leptin production and induces a rapid elevation in levels of anti-inflammatory substances, including IL-1 and IL-10 [46]. Last but not least, smoke is a trigger factor in the development of autoimmune diseases, in particular the onset of RA. Smoke is able to activate inflammatory status, by increasing [47] local inflammation inducing a raised number of neutrophils, macrophages and markers of oxidative stress and reducing relevant cellular repair mechanisms, and it is associated with increased levels of acute phase proteins and cytokines, including CRP, IL-6 and TNF, which occur secondary to direct effects in activation of microglia and astrocytes [48]. In addition to the common mechanisms of the two diseases, smoking can trigger pathogenetic mechanisms characteristic of individual pathologies. As it was summarized by Chang et al. [49], in RA, cigarette smoking increased the expression of matrix metalloproteinase (MMP)-12 (macrophage elastase), implicated in RA pathogenesis, resulting in severe synovial thickening, pannus formation and prominent macrophage infiltration. Besides, smoke can trigger HLA-DR-restricted immune reactions to autoantigens modified by citrullination with a high risk of developing ACPA. On the other hand, smoking in SpA has been shown to raise levels of CRP and pro-inflammatory cytokines by altering the flora of the oral mucosal cavity, with development of periodontitis and by modifying the intestinal microbiota, that is implicated in widening the IL-23/Th17 axis response, underlying the development of SpA [50]. In addition, recent studies have shown that cigarette smoke could be the basis of mRNA expression of bone morphogenetic proteins (BMP) in periosteum, resulting in ossification of the vertebral corner and progression of radiographic damage [51]. As regards pharmacological therapies, it is well known that smoker patients have a low response to therapeutic treatments, as TNF-i, and a markedly poor outcome in comparison with non-smoking patients, due to the imbalance between production and elimination of pro-inflammatory cytokines [52]. Recently, it has been demonstrated that pro-inflammatory cytokines are also involved in the perception of pain, which is why acute and chronic pain has become one of the causes of depression in patients with arthritis [53]. Inflammatory response can influence both acute and chronic pain and the development of depression’s sickness behavior: inflammation is related to depression due to the role of TNF and IL-6, as it was previously discussed, meanwhile pain and depression may co-occur because they are affected by the same modulatory neural system [53]. Moreover, comorbid pain and depression lead to higher functional impairments than depression alone. Patients with major depression and chronic pain are found to be 2.1–4.6 times more likely to report impairment of daily activities, family and social functioning compared to patients affected by depression without chronic pain [54].

### Acute and chronic pain

Pain is defined as an unpleasant sensory and emotional experience associated with actual or potential tissue damage or is described in terms of “damage” [55]. Functional imaging studies have shown a bidirectional relationship between pain and depression in which depression is a risk factor for pain and pain a risk factor for depression. Chronic conditions of pain have been associated with alterations in the regions of the brain responsible for the processing of emotional stimuli. Meerwijk et al. [56] have underlined how anterior cingulate cortex, posterior cingulate cortex, thalamus, cerebellum and parahippocampal gyrus are, equally, the areas of the brain most activated during the experience of psychological pain and sadness [57]. Other experimental studies have demonstrated that peripheral induced pain is associated with high levels of IL-1 and TNF in the cingulate cortex, and an increased expression of inflammatory genes in the amygdala may induce neuropathic pain [58].

An overlap between pain development and depression was also evident with regard to neurochemical markers [59]: pain and mood are both controlled by common neurotransmitters such as serotonin, norepinephrine and glutamate, whose homeostasis varies as a result of previously explained inflammatory processes.

Regarding the association between inflammation and pain, inflammatory mediators are thought to play a key role in the occurrence of hyperalgesia: TNF, linked to TNF-receptors in the dorsal root ganglia, is able to induce peripheral pain in the corresponding innervation area [60].

As a means of determining these observations, clinical trials are ongoing in order to evaluate the use of antidepressant drugs in chronic pain control [61].

### Quality of life

There are 3 factors affecting the quality of life, currently identified in rheumatological patients, related to the appearance of depressive states: pain, somatization and disability.

The topic of pain has already been addressed, underlining how it is partly generated by the involvement of the same neurotransmitters and pro-inflammatory cytokines that regulate the development of depression. In addition, depression in patients with arthritis can exacerbate pain [62], meanwhile the treatment of pain can reduce the symptoms of depression [61]. The somatization, understood as a large number of reported bodily symptoms regardless of their cause [63], is considered a powerful predictor of health-related quality of life (HRQoL) in patients with medically unexplained symptoms and chronic diseases [64], such as arthritis in rheumatological conditions. It could also constitute an important risk factor for the occurrence of depressive and anxiety disorders and represents a factor of minor improvement of depressive and anxiety symptoms, and a predictor of a poorer treatment response in patients [65].

Several mechanisms have been proposed to explain how these conditions are related [66]:

- Depression and anxiety may be a reaction to somatization;
- Somatization may be part of, or a consequence of depression and anxiety;
- That all these conditions are simply different expressions and dimensions of a common underlying form of distress.

There are no definitive answers to this yet. Actually, an association between somatization, depression and chronic pathological conditions has been supposed. Therefore, it is necessary to carry out new studies aimed at identifying this association and discovering the deepest common pathogenetic mechanisms. As regards disability, it is a phenomenon that most rheumatological patients have in common, regardless of the diagnosis [67]. Each rheumatological disease may, in fact, be responsible for the involvement of one or more organs, forcing the patient to reorganize his or her existence. Therefore, over and above the problem of pain, disability is able to modify the quality of life because it modifies daily life itself and reduces personal independence [68]. Several studies have been carried out over the years and have shown how disability is one of the main factors with a negative influence on the quality of life, regardless of the presence or absence of pain. Margaretten et al. [69], for example, evaluated the predictors of depression in a pool of RA patients, highlighting how, in the Caucasian population, disability was the element most influencing quality of life. Similarly, the study conducted by Hyphantis et al. [63] showed that in rheumatologic diseases (where arthritis-related pain as one of the core symptoms of the diseases) somatization and disability were still related to HRQOL even after pain relief; this is probably due to a combination of the physical effects of the disease process and the individual psychological reaction, such as tendency to worry, previous illness experience and rapid disease progression.

In summary, depression is now a deeply rooted comorbidity in rheumatological patients, so much so that it has become one of the key points that should be taken into account as regards the treat-to-target recommendations in patients with RA and SpA [70, 71].

### Pharmacological therapies

Concerning the pharmacological therapies for rheumatic diseases treatment and their link with depressive syndromes in patients, two main aspects should be taken into consideration:

- How some drugs may be responsible for the onset of depression in rheumatic patients.
- How other pharmacological molecules can improve the mood in patients already diagnosed with depression or depressive disorders.

Regarding the first point, it is mandatory to discuss the role of glucocorticoids in the management of patients with inflammatory diseases. Steroids represent one of the most widely used drugs in the management of these patients, both as regards the treatment of episodes of exacerbation and as chronic underlying treatment. However, over the years the countless side effects that these molecules can produce, especially when used at high dosages and for long periods, have been uncovered, as alterations in mood tone [72]. Yet in the late 1940s, glucocorticoids proved to be responsible for clinical manifestations as severe mood disturbances, psychosis, and even suicide [73]. Several studies have been conducted to try to understand the mechanisms underlying the onset of these symptoms. It has been shown, for example, that in animal models exposure to high levels of corticosteroids is associated with changes in brain tissue: decreased numbers of dendritic branch points and reduced apical dendrite length in the rat hippocampus [74]. Similar results have also been confirmed by studies conducted in humans. In this context, Wilner et al. [75] reported a hippocampal atrophy detected using computerized tomography (CT), associated with an impairment in cognitive tests in patients on corticosteroid therapy, for a period of at least 5 years. Furthermore, Brown et al*.* evaluated memory deficits and brain structure alterations in patients on chronic steroid therapy in several studies, when comparing their performance with a healthy control group [76]. Treated patients, in addition to poorer performance on memory tests when compared to the control group, have a lower hippocampal volume and lower levels of temporal lobe N-acetyl aspartate (a marker of neuronal viability). In addition, a significant correlation was found between the dose of cortisone taken and right hippocampal volume [76]. Finally, in the 4-year follow-up, corticosteroid-treated patients presented clinical stability regarding psychiatric and neurocognitive symptoms, while depressive symptoms had progressively increased [77]. Over and above the aspect of altered volume of the hippocampus, another element that negatively affects the humoral sphere of patients on chronic cortisone therapy is the involvement of hypothalamus–pituitary–adrenal axis. The inflammatory process leads to an alteration to and consequent decrease in inhibitory feedback on CRH by glucocorticoids, resulting in production of TNF and IL-6 and amplification of the inflammatory process [30]. This mechanism is further exacerbated and increased by the chronic introduction of glucocorticoids as treatment. Cortisone also acts on other neurotransmitters: it induces a reduction in cerebral spinal fluid levels of corticotropin, norepinephrine, β-endorphin, β-lipotropin, somatostatin, and an increase in the glutamate release [78], responsible for the processes of cell apoptosis and oxidative damage.

However, drug therapy is not always a reason for the appearance or worsening of depressive symptoms: this is the case of TNF-i.

TNFα represents a molecule whose role in the onset of depression has been widely demonstrated [18]. In this context, it was assumed that drugs directed against this cytokine were able to improve not only inflammatory symptoms, but also those related to depression as has been demonstrated in rodent models, in which administration of TNF-i resulted in a reduction of depression-like and anxiety-like symptoms [79]. So far, some trials have been conducted in order to investigate the role of TNF-i in depressive disorders among rheumatological patients and a complete meta-analysis of all these studies has been carried out by Abbott R and collaborators [80]. This analysis asserts that TNF-i are, actually, only able to reduce depressive symptoms and anxiety crises partially, however, not to the extent expected. In addition, in real-world clinical practice, only a certain percentage of patients has a good clinical response for joint symptoms when treated with TNF-i [80] and it must, therefore, be assumed that the beneficial effect on depression can actually occur only in this percentage of patients. Fiest et al. [81] supported these findings, analyzing 3 studies conducted on the use of biological disease-modifying anti-rheumatic drugs (bDMARDs) in patients suffering from RA and depression, and one, in particular, showed less frequent mood and anxiety disorders in patients treated with TNF-i compared with those on non-biological or no DMARDs therapy. No studies on molecules other than the TNF-i have been performed. Fiest also analyzed the available data from the opposite perspective, trying to understand if the drugs used in the management of patients with depressive syndromes may or may not affect the symptoms and disease activity in patients with RA. However, psychotropic drugs may exacerbate the level of fatigue [82] and may, also, interact with treatments commonly used for depression and DMARDs or other anti-inflammatory and immunomodulatory therapies, such as alteration of coagulation [83].

### Depressive syndromes in rheumatological diseases

As already mentioned, the prevalence of depressive syndromes in patients with inflammatory arthritis is extremely variable, depending on the criteria of diagnosis and evaluation. Certainly, there are many pathogenetic mechanisms common to different diseases, while others are peculiar to an individual disease. Clinical manifestations, as well as the presence of comorbidities, can, also, influence the development of different moods [84]. Accordingly, the clinical manifestations also vary, depending on the rheumatological diseases taken into consideration.

#### Rheumatoid arthritis

Lu et al. [85] carried out a national longitudinal search using the National Health Insurance Research Database of Taiwan, concluding that a bidirectional association between RA and depression exists. Consequently, they aimed at analyzing in RA patients, factors predicting the onset of depression and in patients with depression, factors predicting RA. In support of previous reports, their analysis revealed that a higher risk of depression occurred within 2 years of RA diagnosis and individuals with depression have a high risk of developing RA. This association has, once again, been identified on a physiological and pathogenetic level: neurotransmitter deregulation and dysfunctional intracellular signaling [29], activation of intracellular signaling pathway, such as SAPK/MAPK and PI-3 K/AKT/mTOR [86] and elevated level of pro-inflammatory cytokines emerging from psychiatric disorders that may provoke chronic medical illnesses [18].

Furthermore, Katz et al. [87] evaluated which factors can influence the worsening of fatigue, among the key RA symptoms. Their study has shown that depression is one of the main factors linked to fatigue exacerbation, in association with poor sleep, and that depressed mood and poor sleep were independent predictors of fatigue in RA, independent of disease activity.

Finally, treatment response is, also, reduced in patients with RA and depression, due to its impact on treatment outcome and its influence on the experience of symptoms and health behavior [88]. Depressive symptoms are associated with lower adherence to both pharmacological and non-pharmacological interventions [89]; conversely, a vicious cycle of disease activity, through in amatory cytokines, pain and disability, increased the prevalence and severity of depressive symptoms while effective treatment of RA improves the rate of depression.

#### Spondyloarthritis

SpA is a group of inflammatory diseases of the skeleton characterized by some common points, including the asymmetry of joint involvement, the absence of standardized laboratory markers and the presence of other strongly associated clinical conditions such as skin psoriasis. It is possible to distinguish between peripheral forms, with arthritis developing mainly in the small joints of the limbs, and axial forms (axSpA), mainly involving the spine and the sacroiliac joints. In the latter group it is possible, using diagnostic imaging methods (non-radiographic axSpA or nr-axSpA), to make a further distinction between axSpA with evidence of radiographic damage and axSpA where joint damage is not yet detectable. Like other conditions for which chronic pain is the main clinical feature, axSpA are associated with depression [5, 10]. However, quantifying the prevalence of depression is challenging, mainly due to the distinction of AS and nr-axSpA [12].

The analysis conducted by Strand et al. in 2017 highlights relevant items underlying the development of depression in patients suffering from SpA [90]. First of all, persistent inflammation of the sacroiliac joints causes chronic inflammatory back pain and persistent symptoms of stiffness and fatigue. Moreover, the progressive bony fusion of the spine is a major contributor to disease burden and limits physical functionality, including the ability to perform activities of daily life and social interactions. Strand points out that SpA patients present high or moderate work instability, such as impaired relationships with intimate partners, due to spinal deformation/curvature, poor posture, lower urinary tract symptoms and sexual dysfunction [90]. In addition, as a result of the significant body image disturbances, SpA patients, also, suffer from anxiety and depression [91].

#### Psoriatic arthritis

Inflammatory mechanisms, related to the development of symptoms such as pain and fatigue, which, in turn, are responsible for the development of depressive symptoms, are present not only in RA, but also in patients affected by PsA. However, unlike RA, in PsA part of the development of mood disorders is due to the concomitant skin involvement along with the presence of numerous comorbidities [92]. As regards skin involvement, studies have shown that patients with skin psoriasis, in visible areas, suffer more from poor psychosocial function due to public embarrassment. In particular, patients feel stigmatized by the disease, reporting feelings of rejection, shame, and guilt. These feelings manifest not only in work and social interactions, but also as sexual dysfunction, especially when the genitals are involved [92]. This results not only in an increased predisposition to the development of depressive disorders, but also in suicidal ideation reported in approximately 10% of young patients aged 18–34 years [93]. In addition, the presence of comorbidities negatively influences a PsA patient's mood and eventual quality of life. It is estimated that about 50% of PsA patients suffer from at least 1 comorbidity, the greatest of which are cardiovascular disease, ophthalmic disease, liver disease, inflammatory bowel disease [94]; when compared to other cohorts, such as patients with psoriasis alone, PsA patients have significantly poorer quality of life and a higher incidence of depression and anxiety [95].

## Discussion and highlights

**Fig. 1:**
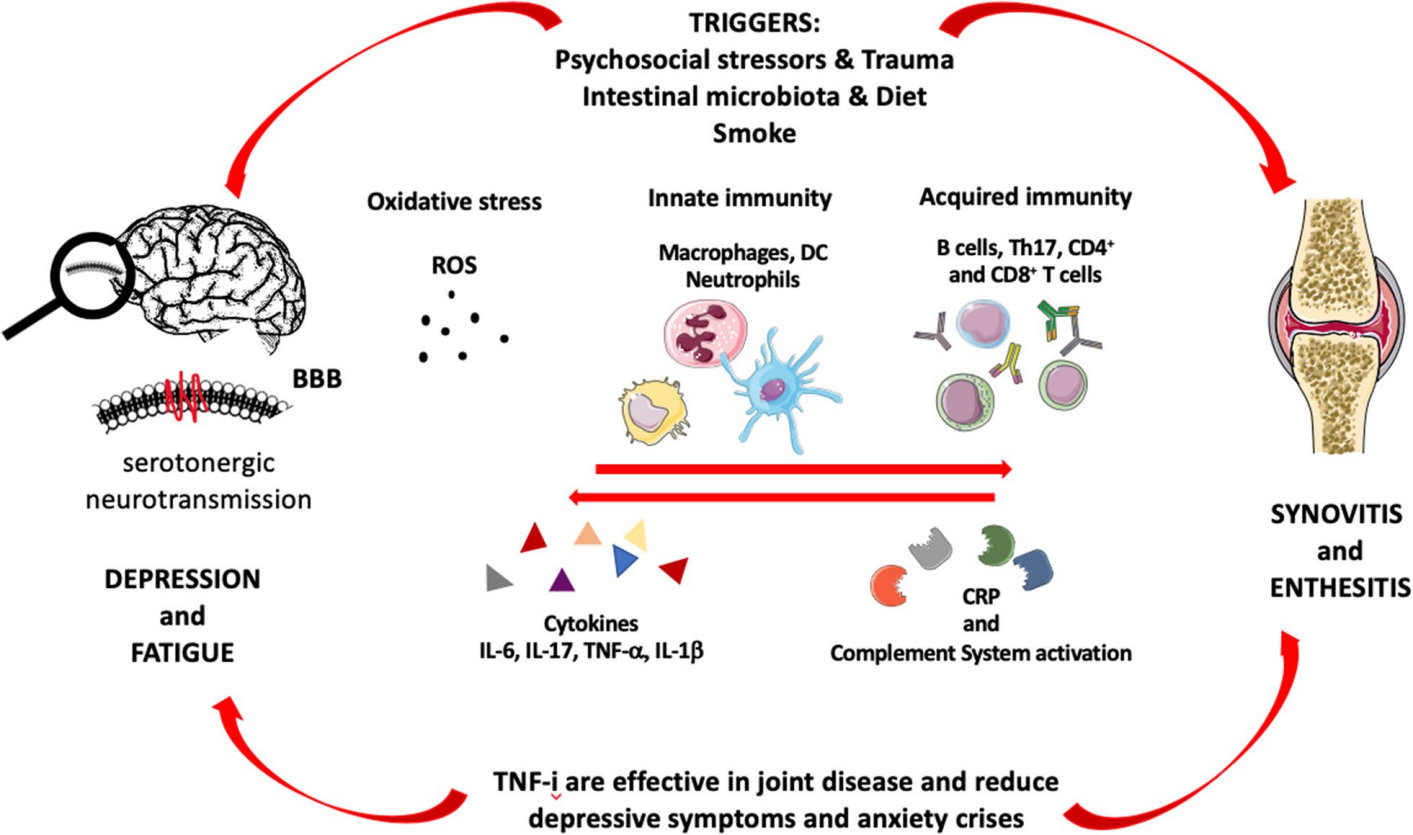
Crosstalk between brain and joint: pathogenetic pathways involved in both depressive syndromes and inflammatory joint diseases. *BBB* blood–brain barrier, *ROS* reactive oxygen species, *DC* dendritic cells, *IL* interleukin, *TNF* tumor necrosis factor, *CRP* C-reactive protein

Depressive disorders are described in patients affected by inflammatory arthritis [5]. Significant studies summarized in this review are presented in Table 2. Crosstalk between brain and joints is summarized in Fig. 1. Estimated prevalence are extremely heterogeneous and their variability depends on the diagnostic criteria as well as the psychometric instruments used to assess the presence or absence of depression [5, 10, 11, 62, 96–98]. It is, however, reasonable to think, weighing all the studies analyzed, that the prevalence is between 15 and 20% of the population affected by inflammatory arthritis. Independently of this, it emerges from all the studies analyzed that depression and mood disorders are, to all intents and purposes, a problem that is widely present in rheumatological patients, so much so, that it can now be counted among the comorbidities that characterize these diseases [13]. As for other concomitant pathologies, such as cardiovascular diseases for example, depressive disorders also developed due to inflammatory pathways and the presence of inflammatory cytokines that may alter neurotransmission and pain perception [18, 29, 30, 55]. In addition, depression and inflammatory arthritis share common symptoms and signs, such as fatigue, asthenia, poor sleep, daily disability and alterations in social interaction, as well as disorders of the body image and the sexual sphere [87, 94, 99]. As regards the characteristics of the population most affected, a great variability, depending on the rheumatological disease, must be considered, its prevalence in the general population, and the type of depressive symptom considered. For example, it has been demonstrated that in RA patients depression usually occurs 2 years after diagnosis and mainly affects women aged 50 years [5]. This data dramatically changes if SpA patients are considered, where men are the most affected, especially in relation to urinary tract and sexual function problems [91].

**Table 2:** Studies included for qualitative synthesis

Authors	Population	Intervention	Outcomes	Findings and results
Fung et al.	466 healthy men	Questionnaire about diet habit and correlation with other factors	FFQs	Major dietary patterns are predictors of plasma biomarkers of CVD and obesity risk
Shah et al.	1692 adult patients with inflammatory arthritis	Retrospective longitudinal cohort study evaluating the effect of depressive treatment on pain	SF-12v2, MCS, PCS	Depression treatment didn’t improve pain and health-related quality of life among adults with arthritis
Apfelbacher et al.	Adult patients with inflammatory arthritis	Cross-sectional data from the 2002 World Health Survey	Self-reported depression	Positive association between inflammatory arthritis and depression in Western and Non-Western countries, suggesting that this relationship represents a universal phenomenon
Matcham et al.	56 RA patients	One-year prospective study	HADS	Symptoms of depression and anxiety have implications for disease activity
Margaretten et al.	172 RA patients	Evaluation of depression and health outcome	HAQ, PHQ-9, DAS-28	Higher HAQ scores were associated with depression
Katz et al.	158 RA patients	Cross-sectional study on sources of fatigue in RA patients	Assessments of self-reported sleep quality, depression, physical activity, RA disease activity, muscle strength, functional limitations, body composition; information on demographics, medications, and smoking; the FSI	Fatigue in RA patients is a result of RA disease activity, pain, inactivity, depression, obesity and poor sleep
Lu et al.	8831 RA patients and 15,456 health controls	14-year follow-up nationwide longitudinal study on bidirectional relationships between RA and depression	Demographic variables, urbanization level, baseline comorbidities, incidence of depression	A strong bidirectional relationships between RA and depression
Figueiredo-Braga et al.	82 RA patients, 73 SLE, 22 healthy subjects and 32 depressed control subjects	Cross-sectional study	FSS, HADS, PSQI, RAS, DAS28	IL-10 and IL-6 are associated with depressive symptoms
Hyphantis et al.	524 patients affected by RA, SLE, SSc, Glaucoma and Colon cancer	Cross-sectional study to test the relative importance of depression in HRQOL in several chronic physical disorders	SCL-90 and WHO Quality of Life Instrument Short Form, HRQOL	SCL-90 somatization score significantly correlated to physical HRQOL in all diseases

*RA* rheumatoid arthritis, *PHQ-9* Patient Health Questionnaire-9, *HADS* Hospital Anxiety and Depression Scale, *FFQs* food-frequency questionnaires, *CVD* cardiovascular diseases, *MR* magnetic resonance, *HAQ* Health Assessment Questionnaire, *DAS-28* Disease Activity Score 28, *SCL-90* Symptom Distress Checklist, *SLE* systemic lupus erythematosus, *SSc* systemic sclerosis, *HRQoL* Health-Related Quality of Life, *PsA* psoriatic arthritis, *SpA* spondyloarthritis, *TNF-*α tumor necrosis factor α, *SMDs* standardized mean differences, *FSI* Fatigue Severity Inventory, *AxSpA* axial spondyloarthritis, *SF-12v2* Short Form Health Survey-12 version two, *MCS* Mental Component Summary Score, *PCS* Physical Component Summary Score, *FSS* Fatigue Severity Scale, *HADS* Hospital Anxiety and Depression Scale, *PSQI* Pittsburgh Sleep Quality Index, *RAS* Relationship Assessment Scale, *HADS* Hospital Anxiety Depression Scale

Another missing aspect in the studies, certainly representing a reality to face in daily clinical practice, is the concomitant presence in some patients with arthritis of a condition of fibromyalgia (FM) [100]. FM, defined as an idiopathic syndrome causing an increase in muscle tension, shares numerous symptoms with both depressive disorder and inflammatory arthritis, such as chronic pain, stiffness, fatigue, insomnia and sleep disorders [101]. FM patients present a decrease in serotonin levels, which may lead to the development of anxiety and depressive disorders [102]. How can depression as comorbidity and depression as a consequence of a concomitant FM syndrome be distinguish? There are currently few answers, but none are satisfactory. Further prospective studies are, therefore, necessary to evaluate this important aspect of rheumatology and allowing physicians, in the future, not only to differentiate the type of rheumatological patient, but also to start a specific treatment focused on the problem to be addressed.

Finally, a short focus on therapies. Studies conducted on the use of TNF-i for the treatment of joint disease and comorbid depressive symptoms were considered. The data are scarce, partially inconsistent, and certainly not comforting. In fact, the results show that TNF-i are only partially effective in softening depressive and anxiety symptoms in patients who have an adequate clinical response regarding joint symptoms [80]. On the other hand, psychotropic drugs used in the management of patients may exacerbate the level of fatigue [82] and interact with treatments commonly used for arthritis management, such as NSAIDs and DMARDs [83].

## Conclusion

Studies considered highlighted an intriguing relationship between inflammatory rheumatological diseases and depression. This relationship is related either to the causes of the co-occurrence of the diseases or their similar clinical pattern. However, additional important shortcomings in the evidence of the prevalence of depression in inflammatory arthritis, need to be addressed. Symptoms of depression and anxiety have several implications on disease activity, primarily due to their influence on tender joints and on patient global assessment. The knowledge of the common pathogenetic mechanisms among the two disorders should have implications for treatment decision-making in inflammatory diseases. Inflammatory markers may indicate significant psychological morbidity and related non-inflammatory pain, rather than true disease activity. There are still too few studies analyzing the burden of depression in rheumatological patients, in particular in patients with PsA and SpA, and they are extremely heterogeneous both in the characteristics of the population taken into consideration and in the methods used to treat depressive disorders. No studies on fibromyalgia syndrome are, as yet, available.

## Data Availability

The dataset is available from the corresponding author on reasonable request.

## References

[1] Chimenti MS, Triggianese P, De Martino E, Conigliaro P, Fonti GL, Sunzini F, Caso F, Perricone C, Costa L, Perricone R (2019). An update on pathogenesis of psoriatic arthritis and potential therapeutic targets. Expert Rev Clin Immunol.

[2] Conigliaro P, Triggianese P, De Martino E, Fonti GL, Chimenti MS, Sunzini F, Viola A, Canofari C, Perricone R (2019). Challenges in the treatment of Rheumatoid Arthritis. Autoimmun Rev.

[3] Conigliaro P, Chimenti MS, Triggianese P, Sunzini F, Novelli L, Perricone C, Perricone R (2016). Autoantibodies in inflammatory arthritis. Autoimmun Rev.

[4] Chimenti MS, Caso F, Alivernini S, Costa L, Tolusso B, Triggianese P, Conigliaro P, Gremese E, Scarpa R, Perricone R (2019). Amplifying the concept of psoriatic arthritis: the role of autoimmunity in systemic psoriatic disease. Autoimmun Rev.

[5] Matcham F, Rayner L, Steer S, Hotopf M (2013). The prevalence of depression in rheumatoid arthritis: a systematic review and meta-analysis. Rheumatology (Oxford).

[6] Lok EYC, Mok CC, Cheng CW, Cheung EF (2010). Prevalence and determinants of psychiatric disorders in patients with rheumatoid arthritis. Psychosomatics.

[7] Isik A, Koca SS, Ozturk A, Mermi O (2007). Anxiety and depression in patients with rheumatoid arthritis. Clin Rheumatol.

[8] Imperatori C, Bianciardi E, Niolu C, Fabbricatore M, Gentileschi P, Di Lorenzo G, Siracusano A, Innamorati M (2020). The symptom-checklist-K-9 (SCL-K-9) discriminates between overweight/obese patients with and without significant binge eating pathology: psychometric properties of an Italian version. Nutrients.

[9] Huang X, Magder LS, Petri M (2014). Predictors of incident depression in systemic lupus erythematosus. J Rheumatol.

[10] Esposito M, Saraceno R, Giunta A, Maccarone M, Chimenti S (2006). An Italian study on psoriasis and depression. Dermatology.

[11] Zhao S, Thong D, Miller N, Duffield SJ, Hughes DM, Chadwick L, Goodson NJ (2018). The prevalence of depression in axial spondyloarthritis and its association with disease activity: a systematic review and meta-analysis. Arthritis Res Ther.

[12] Dantzer R, O’connor JC, Freund GG, Johnson RW, Kelley KW (2008). From inflammation to sickness and depression: when the immune system subjugates the brain. Nat Rev Neurosci..

[13] Dantzer R (2004). Cytokine-induced sickness behaviour: a neuroimmune response to activation of innate immunity. Eur J Pharmacol.

[14] Lee CH, Giuliani F (2019). The role of inflammation in depression and fatigue. Front Immunol.

[15] Zorrilla EP, Luborsky L, Mckay JR, Rosenthal R, Houldin A, Tax A, McCorkle R, Seligman DA, Schmidt K (2001). The relationship of depression and stressors to immunological assays: a meta-analytic review. Brain Behav Immun.

[16] Sautner J, Puchner R, Alkin A, Pieringer H (2020). Depression: a common comorbidity in women with rheumatoid arthritis—results from an Austrian cross-sectional study. BMJ Open.

[17] Pryce CR, Fontana A (2017). Depression in autoimmune diseases. Curr Top Behav Neurosci.

[18] Beurel E, Lowell JA (2018). Th17 cells in depression. Brain Behav Immun.

[19] Amatya N, Garg AV, Gaffen SL (2017). IL-17 Signaling: the Yin and the Yang. Trends Immunol.

[20] Zimmermann J, Krauthausen M, Hofer MJ, Heneka MT, Campbell IL, Muller M (2013). CNS-targeted production of IL-17A induces glial activation, microvascular pathology and enhances the neuroinflammatory response to systemic endotoxemia. PLoS ONE.

[21] Moynes DM, Vanner SJ, Lomax AE (2014). Participation of interleukin 17A in neuroimmune interactions. Brain Behav Immun.

[22] Gaffen SL (2009). Structure and signalling in the IL-17 receptor family. Nat Rev Immunol.

[23] Maes M, Song C, Lin A, De Jongh R, Van Gastel A, Kenis G, Bosmans E, De Meester I, Benoy I, Neels H, Demedts P, Janca A, Scharpé S, Smith RS (1998). The effects of psychological stress on humans: increased production of pro-inflammatory cytokines and a Th1-like response in stress-induced anxiety. Cytokine.

[24] Dantzer R, O'Connor JC, Freund GG, Johnson RW, Kelley KW (2008). From inflammation to sickness and depression: when the immune system subjugates the brain. Nat Rev Neurosci.

[25] Lorton D, Lubahn CL, Zautra AJ, Bellinger DL (2008). Proinflammatory cytokines and sickness behavior in rheumatic diseases. Curr Pharm Des.

[26] Lorton D, Lubahn CL, Estus C, Millar BA, Carter JL, Wood CA, Bellinger DL (2006). Bidirectional communication between the brain and the immune system: implications for physiological sleep and disorders with disrupted sleep. NeuroImmunoModulation.

[27] McCusker RH, Kelley KW (2013). Immune-neural connections: how the immune system's response to infectious agents influences behavior. J Exp Biol.

[28] Shearer WT, Reuben JM, Mullington JM, Price NJ, Lee BN, Smith EO, Szuba MP, Van Dongen HP, Dinges DF (2001). Soluble TNF-alpha receptor 1 and IL-6 plasma levels in humans subjected to the sleep deprivation model of spaceflight. J Allergy Clin Immunol.

[29] Ruhe HG, Mason NS, Schene AH (2007). Mood is indirectly related to serotonin, norepinephrine and dopamine levels in humans: a meta-analysis of monoamine depletion studies. Mol Psychiatry.

[30] Pariante CM (2003). Depression, stress and the adrenal axis. J Neuroendocrinol.

[31] Bianciardi E, Fabbricatore M, Di Lorenzo G, Innamorati M, Tomassini L, Gentileschi P, Niolu C, Siracusano A, Imperatori C (2019). Prevalence of food addiction and binge eating in an Italian sample of bariatric surgery candidates and overweight/obese patients seeking low-energy-diet therapy. Riv Psichiatr.

[32] Perrone F, Bianciardi E, Benavoli D, Tognoni V, Niolu C, Siracusano A, Gaspari AL, Gentileschi P (2016). Gender influence on long-term weight loss and comorbidities after laparoscopic sleeve gastrectomy and Roux-en-Y gastric bypass: a prospective study with a 5-year follow-up. Obes Surg.

[33] Fung TT, Rimm EB, Spiegelman D, Rifai N, Tofler GH, Willett WC, Hu FB (2001). Association between dietary patterns and plasma biomarkers of obesity and cardiovascular disease risk. Am J Clin Nutr.

[34] Chrysohoou C, Panagiotakos DB, Pitsavos C, Das UN, Stefanadis C (2004). Adherence to the Mediterranean diet attenuates inflammation and coagulation process in healthy adults: the ATTICA Study. J Am Coll Cardiol.

[35] Caso F, Navarini L, Carubbi F (2020). Mediterranean diet and Psoriatic Arthritis activity: a multicenter cross-sectional study. Rheumatol Int.

[36] Volman JJ, Ramakers JD, Plat J (2008). Dietary modulation of immune function by beta-glucans. Physiol Behav.

[37] Chimenti MS, Perricone C, Novelli L (2018). Interaction between microbiome and host genetics in psoriatic arthritis. Autoimmun Rev.

[38] Rangel-Huerta OD, Aguilera CM, Mesa MD, Gil A (2012). Omega-3 long-chain polyunsaturated fatty acids supplementation on inflammatory biomarkers: a systematic review of randomized clinical trials. Br J Nutr.

[39] Iwata NG, Pham M, Rizzo NO, Cheng AM, Maloney E, Kim F (2011). Trans fatty acids induce vascular inflammation and reduce vascular nitric oxide production in endothelial cells. PLoS ONE.

[40] Beilfuss J, Berg V, Sneve M, Jorde R, Kamycheva E (2012). Effects of a 1-year supplementation with cholecalciferol on interleukin-6, tumor necrosis factor-alpha and insulin resistance in overweight and obese subjects. Cytokine.

[41] Sampogna G, Fiorillo A, Luciano M, Del Vecchio V, Steardo L, Pocai B, Barone M, Amore M, Pacitti F, Dell'Osso L, Di Lorenzo G, Maj M, LIFESTYLE Working Group (2018). A randomized controlled trial on the efficacy of a psychosocial behavioral intervention to improve the lifestyle of patients with severe mental disorders: study protocol. Front Psychiatry..

[42] Bianciardi E, Gentileschi P, Niolu C, Innamorati M, Fabbricatore M, Contini LM, Procenesi L, Siracusano A, Imperatori C (2020). Assessing psychopathology in bariatric surgery candidates: discriminant validity of the SCL-90-R and SCL-K-9 in a large sample of patients. Eat Weight Disord.

[43] Izaola O, de Luis D, Sajoux I, Domingo JC, Vidal M (2015). Inflammation and obesity (lipoinflammation). Nutr Hosp.

[44] Montuori M, Benavoli D, D'Ugo S, Di Benedetto L, Bianciardi E, Gaspari AL, Gentileschi P (2017). Integrated approaches for the management of staple line leaks following sleeve gastrectomy. J Obes.

[45] Bano G, Trevisan C, Carraro S, Solmi M, Luchini C, Stubbs B, Manzato E, Sergi G, Veronese N (2017). Inflammation and sarcopenia: a systematic review and meta-analysis. Maturitas.

[46] Paolucci EM, Loukov D, Bowdish DME, Heisz JJ (2018). Exercise reduces depression and inflammation but intensity matters. Biol Psychol.

[47] van der Vaart H, Postma DS, Timens W, ten Hacken NH (2004). Acute effects of cigarette smoke on inflammation and oxidative stress: a review. Thorax.

[48] Batatinha HAP, Rosa Neto JC, Krüger K (2019). Inflammatory features of obesity and smoke exposure and the immunologic effects of exercise. Exerc Immunol Rev.

[49] Chang K, Yang SM, Kim SH, Han KH, Park SJ, Shin JI (2014). Smoking and rheumatoid arthritis. Int J Mo Sci.

[50] Wang H, Peng W, Weng Y (2012). Imbalance of Th17/Treg cells in mice with chronic cigarette smoke exposure. Int Immunopharmacol.

[51] Chassanidis CG, Malizos KN, Varitimidis S, Samara S, Koromila T, Kollia P, Dailiana Z (2012). Smoking affects mRNA expression of bone morphogenetic proteins in human periosteum. J Bone Joint Surg Br.

[52] Abhishek A, Butt S, Gadsby K, Zhang W, Deighton CM (2010). Anti-TNF-alpha agents are less effective for the treatment of rheumatoid arthritis in current smokers. J Clin Rheumatol.

[53] Doan L, Manders T, Wang J (2015). Neuroplasticity underlying the comorbidity of pain and depression. Neural Plast.

[54] Ohayon MM, Schatzberg AF (2010). Chronic pain and major depressive disorder in the general population. J Psychiatr Res.

[55] Treede RD (2018). The International Association for the Study of Pain definition of pain: as valid in 2018 as in 1979, but in need of regularly update footnotes. Pain Rep.

[56] Meerwijk EL, Ford JM, Weiss SJ (2013). Brain regions associated with psychological pain: implications for a neural network and its relationship to physical pain. Brain Imaging Behav.

[57] Shirai M, Soshi T (2019). Why is heartache associated with sadness? Sadness is represented by specific physical pain through verbal knowledge. PLoS ONE.

[58] Lu Y, Zhu L, Gao YJ (2011). Pain-related aversion induces astrocytic reaction and proinflammatory cytokine expression in the anterior cingulated cortex in rats. Brain Res Bull.

[59] Leonard BE (2015). Pain, depression and inflammation: are interconnected causative factors involved?. Mod Trends Pharmacopsychiatry.

[60] Boettger MK (2008). Antinociceptive effects of tumour ne- crosis factor alpha neutralisation in a rat model of antigen-induced arthritis: evidence of a neuronal target. Arthritis Rheum.

[61] Khouzam HR (2016). Psychopharmacology of chronic pain: a focus on antidepressants and atypical antipsychotics. Postgrad Med.

[62] Shah D, Rai P, Dwibedi N, Sambamoorthi U (2018). Treatment for depression and health-related quality of life among adults with arthritis. Psychiatr Q.

[63] Hyphantis T, Tomenson B, Paika V, Almyroudi A, Pappa C, Tsifetaki N, Voulgari PV, Drosos AA, Pavlidis N, Creed F (2009). Somatization is associated with physical health-related quality of life independent of anxiety and depression in cancer, glaucoma and rheumatological disorders. Qual Life Res.

[64] Feder A, Olfson M, Gameroff M, Fuentes M, Shea S, Lantigua RA, Weissamn MM (2001). Medically unexplained symptoms in an urban general medicine practice. Psychosomatics.

[65] Huijbregts KM, van Marwijk HW, de Jong FJ, Schreuders B, Beekman AT, van der Feltz-Cornelis CM (2010). Adverse effects of multiple physical symptoms on the course of depressive and anxiety symptoms in primary care. Psychother Psychosom.

[66] Dijkstra-Kersten SM, Sitnikova K, van Marwijk HW, Gerrits MM, van der Wouden JC, Penninx BW, van der Horst HE, Leone SS (2015). Somatisation as a risk factor for incident depression and anxiety. J Psychosom Res.

[67] Shim EJ, Hahm BJ, Go DJ, Lee KM, Noh HL, Park SH, Song YW (2018). Modeling quality of life in patients with rheumatic diseases: the role of pain catastrophizing, fear-avoidance beliefs, physical disability, and depression. Disabil Rehabil.

[68] March L, Smith EU, Hoy DG, Cross MJ, Sanchez-Riera L, Blyth F, Buchbinder R, Vos T, Woolf AD (2014). Burden of disability due to musculoskeletal (MSK) disorders. Best Pract Res Clin Rheumatol.

[69] Margaretten M, Yelin E, Imboden J, Graf J, Barton J, Katz P, Julian L (2009). Predictors of depression in a multiethnic cohort of patients with rheumatoid arthritis. Arthritis Rheum..

[70] Smolen JS, Breedveld FC, Burmester GR, Bykerk V, Dougados M, Emery P, Kvien TK, Navarro-Compán MV, Oliver S, Schoels M, Scholte-Voshaar M, Stamm T, Stoffer M, Takeuchi T, Aletaha D, Andreu JL, Aringer M, Bergman M, Betteridge N, Bijlsma H, Burkhardt H, Cardiel M, Combe B, Durez P, Fonseca JE, Gibofsky A, Gomez-Reino JJ, Graninger W, Hannonen P, Haraoui B, Kouloumas M, Landewe R, Martin-Mola E, Nash P, Ostergaard M, Östör A, Richards P, Sokka-Isler T, Thorne C, Tzioufas AG, van Vollenhoven R, de Wit M, van der Heijde D (2016). Treating rheumatoid arthritis to target: 2014 update of the recommendations of an international task force. Ann Rheum Dis.

[71] Smolen JS, Braun J, Dougados M, Emery P, Fitzgerald O, Helliwell P, Kavanaugh A, Kvien TK, Landewé R, Luger T, Mease P, Olivieri I, Reveille J, Ritchlin C, Rudwaleit M, Schoels M, Sieper J, Wit MD, Baraliakos X, Betteridge N, Burgos-Vargas R, Collantes-Estevez E, Deodhar A, Elewaut D, Gossec L, Jongkees M, Maccarone M, Redlich K, van den Bosch F, Wei JC, Winthrop K, van der Heijde D (2014). Treating spondyloarthritis, including ankylosing spondylitis and psoriatic arthritis, to target: recommendations of an international task force. Ann Rheum Dis..

[72] Grennan D, Wang S (2019). Steroid side effects. JAMA.

[73] Niolu C, Bianciardi E, Di Lorenzo G, Nicolai S, Celi M, Ribolsi M, Pietropolli A, Ticconi C, Tarantino U, Siracusano A (2016). Insecure attachment style predicts low bone mineral density in postmenopausal women. A pilot study. Riv Psichiatr.

[74] Zhao Y, Ma R, Shen J, Su H, Xing D, Du L (2008). A mouse model of depression induced by repeated corticosterone injections. Eur J Pharmacol.

[75] Wilner AP, de Varennes B, Gregoire PA, Lupien S, Pruessner JC (2002). Glucocorticoids and hippocampal atrophy after heart transplantation. Ann Thorac Surg.

[76] Brown ES, Woolston J, Frol A, Bobadilla L, Khan DA, Hanczyc M, Rush AJ, Fleckenstein J, Babcock E, Cullum CM (2004). Hippocampal volume, spectroscopy, cognition, and mood in patients receiving corticosteroid therapy. Biol Psychiatry.

[77] Brown ES, Vera E, Frol AB, Woolston DJ, Johnson B (2007). Effects of chronic prednisone therapy on mood and memory. J Affect Disord.

[78] Lin X, Zhao Y, Li S (2017). Astaxanthin attenuates glutamate-induced apoptosis via inhibition of calcium influx and endoplasmic reticulum stress. Eur J Pharmacol.

[79] Krugel U, Fischer J, Radicke S, Sack U, Himmerich H (2013). Antidepressant effects of TNF-alpha blockade in an animal model of depression. J Psychiatr Res.

[80] Abbott R, Whear R, Nikolaou V, Bethel A, Coon JT, Stein K, Dickens C (2015). Tumour necrosis factor-α inhibitor therapy in chronic physical illness: a systematic review and meta-analysis of the effect on depression and anxiety. J Psychosom Res.

[81] Fiest KM, Hitchon CA, Bernstein CN, Peschken CA, Walker JR, Graff LA, Zarychanski R, Abou-Setta A, Patten SB, Sareen J, Bolton J, Marrie RA (2017). CIHR Team “defining the burden and managing the effects of psychiatric comorbidity in chronic immunoinflammatory disease” systematic review and meta-analysis of interventions for depression and anxiety in persons with rheumatoid arthritis. J Clin Rheumatol..

[82] Ferguson J (2001). SSRI antidepressant medications: adverse effects and tolerability. Prim Care Companion J Clin Psychiatry.

[83] Lexi-Comp Online. NSAID (nonselective)/antidepressants (tricyclic, tertiary amine). Lexi-Comp Online Interaction Monograph. Hudson, Ohio: Lexi-Comp, Inc; 2015.

[84] Bianciardi E, Vito C, Betrò S, De Stefano A, Siracusano A, Niolu C (2020). The anxious aspects of insecure attachment styles are associated with depression either in pregnancy or in the postpartum period. Ann Gen Psychiatry.

[85] Lu MC, Guo HR, Lin MC, Livneh H, Lai NS, Tsai TY (2016). Bidirectional associations between rheumatoid arthritis and depression: a nationwide longitudinal study. Sci Rep.

[86] Malemud CJ, Miller AH (2008). Pro-inflammatory cytokine-induced SAPK/MAPK and JAK/STAT in rheumatoid arthritis and the new anti-depression drugs. Expert Opin Ther Targets.

[87] Katz P, Margaretten M, Trupin L, Schmajuk G, Yazdany J, Yelin E (2016). Role of sleep disturbance, depression, obesity, and physical inactivity in fatigue in rheumatoid arthritis. Arthritis Care Res (Hoboken).

[88] Dimatteo MR, Lepper HS, Croghan TW (2000). Depression is a risk factor for noncompliance with medical treatment: meta-analysis of the effects of anxiety and depression on patient adherence. Arch Intern Med.

[89] Niolu C, Barone Y, Bianciardi E, Ribolsi M, Marchetta C, Robone C, Ambrosio A, Sarchiola L, Reggiardo G, Lorenzo GD, Siracusano A (2015). Predictors of poor adherence to treatment in inpatients with bipolar and psychotic spectrum disorders. Riv Psichiatr.

[90] Strand V, Singh JA (2017). Patient burden of axial spondyloarthritis. J Clin Rheumatol.

[91] Shen B, Zhang A, Liu J, Da Z, Xu X, Liu H, Li L, Gu Z (2014). Body image disturbance and quality of life in Chinese patients with ankylosing spondylitis. Psychol Psychother.

[92] Husni ME, Merola JF, Davin S (2017). The psychosocial burden of psoriatic arthritis. Semin Arthritis Rheum.

[93] Krueger G, Koo J, Lebwohl M, Menter A, Stern RS, Rolstad T (2001). The impact of psoriasis on quality of life: results of a 1998 National Psoriasis Foundation patient-membership survey. Arch Dermatol.

[94] Husni ME (2015). Comorbidities in psoriatic arthritis. Rheum Dis Clin North Am.

[95] Salaffi F, Carotti M, Gasparini S, Intorcia M, Grassi W (2009). The health-related quality of life in rheumatoid arthritis, ankylosing spondylitis, and psoriatic arthritis: a comparison with a selected sample of healthy people. Health Qual Life Outcomes.

[96] Figueiredo-Braga M, Cornaby C, Cortez A, Bernardes M, Terroso G, Figueiredo M, Mesquita CDS, Costa L, Poole BD (2018). Influence of biological therapeutics, cytokines, and disease activity on depression in rheumatoid arthritis. J Immunol Res.

[97] Apfelbacher C, Brandstetter S, Herr R, Ehrenstein B, Loerbroks A (2017). Depression and inflammatory arthritis are associated in both Western and Non-Western countries: findings from the World Health Survey 2002. J Psychosom Res.

[98] Matcham F, Ali S, Irving K, Hotopf M, Chalder T (2016). Are depression and anxiety associated with disease activity in rheumatoid arthritis? A prospective study. BMC Musculoskelet Disord.

[99] Bianciardi E, Di Lorenzo G, Niolu C, Betrò S, Zerbin F, Gentileschi P, Siracusano A (2019). Body image dissatisfaction in individuals with obesity seeking bariatric surgery: exploring the burden of new mediating factors. Riv Psichiatr.

[100] Duffield SJ, Miller N, Zhao S, Goodson NJ (2018). Concomitant fibromyalgia complicating chronic inflammatory arthritis: a systematic review and meta-analysis. Rheumatology (Oxford).

[101] Clauw DJ (2014). Fibromyalgia: a clinical review. JAMA.

[102] Welsch P, Üçeyler N, Klose P, Walitt B, Häuser W (2018). Serotonin and noradrenaline reuptake inhibitors (SNRIs) for fibromyalgia. Cochrane Database Syst Rev..

